# Purification and Characterization of Plantaricin YKX and Assessment of Its Inhibitory Activity Against *Alicyclobacillus* spp.

**DOI:** 10.3389/fmicb.2021.783266

**Published:** 2021-12-09

**Authors:** Jinjin Pei, Wengang Jin, Jinze Wang, Yigang Huang, Xinsheng Li, Hongxia Zhang, Yonggui Zhang, Amer Ramadan, A. M. Abd El-Aty

**Affiliations:** ^1^Qinba State Key Laboratory of Biological Resources and Ecological Environment, 2011 QinLing-Bashan Mountains Bioresources Comprehensive Development C. I. C., Shaanxi Province Key Laboratory of Bio-Resources, College of Bioscience and Bioengineering, Shaanxi University of Technology, Hanzhong, China; ^2^State Key Laboratory of Biobased Material and Green Papermaking, College of Food Science and Engineering, Qilu University of Technology, Shandong Academy of Science, Jinan, China; ^3^Department of Pharmacology, Faculty of Veterinary Medicine, Cairo University, Giza, Egypt; ^4^Department of Medical Pharmacology, Faculty of Medicine, Atatürk University, Erzurum, Turkey

**Keywords:** bacteriocin, traditional pickles, physicochemical properties, mechanism of action, *Alicyclobacillus* spp.

## Abstract

Consumers prefer natural over synthetic chemical preservatives on a food label. Therefore, it is crucial to ensure the safety and efficacy of such natural preservatives. The emergence of heat-resistant spore-forming *Alicyclobacillus* spp. has been associated with spoilage problems in the fruit juice industry. Herein, a bacteriocin-producing stain YKX was isolated from the traditional pickles in Hanzhong City, China, and it was identified as *Lactobacillus plantarum* by morphological, biochemical, physiological, and genotypic features. A stable bacteriocin, plantaricin YKX, was isolated, purified, and tested for its efficacy against *Alicyclobacillus acidoterrestris*. Plantaricin YKX is a 14-amino acid peptide (Lys-Tyr-Gly-Asn-Gly-Leu-Ser-Arg-Ile-Phe-Ser-Ala-Leu-Lys). Its minimal inhibitory concentrations (MICs) against the tested bacterial and fungal strains were ranged from 16 to 64 μg/mL. It is thermostable and active at pH 3–8. The flow cytometry data and microscopic observations suggested that plantaricin YKX can augment cell membrane permeability, induce potassium ion leakage and pore formation, and disrupt cell membranes. It also affects spore germination and guaiacol production of *A. acidoterrestris*, probably due to upregulation of the *luxS* gene linked to quorum sensing.

## Introduction

Bacteria, such as *Alicyclobacillus* species, spoil foods, juices, carbonated fruit beverages and shelf-stable iced tea (Silva, 2016; Tremarin et al., 2017). Lee et al. (2002) reported that 60% of respondents among 57 companies experienced spoilage incidents, of which 35% were due to *Alicyclobacillus* spp. Various levels of contamination risk exist for diverse juice types, such as apple juice concentrates, 31–75%; orange juice concentrates, 82%; fresh juice, 32%; apple concentrates, 83% (Walker and Phillips, 2008); orange juice, 15%, and fruit concentrate, 8% (Osopale et al., 2017). The principal spoilage characteristic is an off-odor (Walker and Phillips, 2008; Tremarin et al., 2017), ascribed to the metabolites guaiacol and halophenol derivatives 2,6-dibromophenol and 2,6-dichlorophenol (Lee et al., 2002). Thus, spoilage of fruit products by *Alicyclobacillus* is the biggest challenge facing the industry. *Alicyclobacillus* species present a grave threat to the juice industry and other industries producing juice-based beverages, such as carbonated drinks, ice tea or functional drinks, and those containing juice concentrates (Lee et al., 2002; Silva, 2016; Tremarin et al., 2017).

Measures to prevent *Alicyclobacillus* spoilage are essential in the food industry. As the toxicity of chemical preservatives is a concern, there is a particular need for alternative, non-toxic preservatives (Gao et al., 2010; Pei et al., 2017). Bacteriocins from lactic acid bacteria (LAB) are bacterial peptides with low human oral toxicity, making them suitable as food bio-preservatives (Acuna et al., 2012; Pei et al., 2018). LAB bacteriocins can prevent the spoilage of dairy products, bakery goods, beverages, meat, fruits, and seafood (Gálvez et al., 2008; Pei et al., 2020). For instance, the viable counts of *Staphylococcus aureus* were significantly decreased by adding enterocin AS-48 to pumpkin jam stored at 10°C (Viedma et al., 2009). So far, only a few investigations have focused on bacteriocins targeting *Alicyclobacillus* (Viedma et al., 2009; Yue et al., 2013). *Alicyclobacillus*-targeted bacteriocins need to be investigated to inhibit food spoilage and develop novel preservatives.

LAB bacteriocins are typically classified into class I (lanthionine-based, molecular weight (MW) less than 5 kDa) and class II (non-lanthionine-based, MW less than 10 kDa) (Acuna et al., 2012). Class II is further divided into four subclasses: IIa (pediocin-based), IIb (two-peptide), IIc (cyclics), and IId (linear) (Gálvez et al., 2008). Class III is a heat-sensitive macromolecular protein (LHLP) whose molecular weight is generally greater than 10 kDa. Class IV is macromolecular complexes, which contain carbohydrates or lipid groups in addition to proteins. Class II, Class III, and Class IV bacteriocins are named as non-lantibiotic bacteriocins because they do not contain wool sulfur amino acids. Though some bacteriocins have been isolated and characterized (Viedma et al., 2009; Pei et al., 2018), their antibacterial activities are unclear, except for nisin, the typical class I and the most widely used bacteriocin. Nisin inhibits peptidoglycan biosynthesis and forms concrete pores (Wiedemann et al., 2001). A recent study has paid much attention to the inhibitory effects of LAB bacteriocins on biofilm formation (Chopra et al., 2015) because biofilm plays a curial role in food deterioration in the industry.

Herein, we isolated *Lactobacillus plantarum* YKX from traditional pickles in Hanzhong city (China) and purified a bacteriocin, plantaricin YKX, from the cell-free supernatant (CFS) of this strain. Plantaricin YKX displayed potent activity against *Alicyclobacillus* spp. and other spoilage and pathogenic microorganisms. Further, we investigated physicochemical and structural characterizations and mechanism of action of plantaricin YKX against *Alicyclobacillus* spp.

## Materials and Methods

### Microbial Cultures

Lactic acid bacteria were isolated from the traditional pickles in Hanzhong City, Shaanxi Province, China, by an agar streak method. The pickles samples were cut into small pieces and placed in sterilized De Man, Rogosa, Sharpe (MRS) broth (Oxoid, Basingstoke, United Kingdom). After incubation at 37°C for 16 h and reaching the mid-exponential growth phase, one loop was streaked onto an MRS agar plate. After incubation at 37°C for 24 h to the stationary phase, Gram-positive, catalase-negative, and oxidase-negative bacterial strains were chosen as the potential lactic acid bacteria (LAB) (Winkelströter et al., 2015). *Alicyclobacillus* spp. (one strain DSMZ3922 purchased from Deutsche Sammlung von Mikroorganismen und Zellkulturen (DSMZ); 9 strains of *A. acidoterrestris* isolated from apple garden; and three strains of *A. acidocaldarius* isolated from apple juice processing lines by the previous study) (Yue et al., 2013) were cultured in *Alicyclobacillus acidoterrestris* medium (AAM) (Solarbio, Beijing, China) at 45°C for 48 h (Pei et al., 2020). All other bacterial species were maintained on Luria-Bertani (LB) medium (Solarbio, Beijing, China), and fungi were cultured on Potato Dextrose medium (Solarbio, Beijing, China) (Acuna et al., 2012).

### Screening of Bacteriocin-Producing Lactic Acid Bacteria

Bacteriocin-producing LAB strains were isolated and identified through the agar well diffusion technique using *A. acidoterrestris* DSM3922 as an indicator (Yue et al., 2013). Briefly, 1 mL (approximately 10^8^ cells) of indicator strain culture was uniformly diffused into 25 mL of AAM agar and poured into 9-cm plates. After the agar was solidified, wells with a 0.5 mm diameter were cut on the plates. Each isolated colony was cultured in 10 mL of MRS broth at 30°C for 16 h to the mid-exponential phase. The mid-exponential phase cultures were inoculated into 100 mL of fresh MRS medium with an inoculum size of 5% (*v/v*) and then incubated at 30°C for 24 h to the stationary phase to obtain the potential maximum bacteriocin production. As stated elsewhere, the optimum bacteriocin production temperature was lower than the optimum growth temperature. In this study, an incubation temperature of 30°C was used to achieve the maximum bacteriocin production (Viedma et al., 2009; Yue et al., 2013; Pei et al., 2020). Cell-free supernatants were obtained through centrifugation (Avanti J-E, Beckman, California, United States) at 10,000 × *g* for 10 min (4°C), filtered through 0.22-μm pore-size filters (Millipore, MA). Subsequently, 100 μL of the supernatant was inoculated into each well. The plates were incubated at 45°C for 72 h with *Alicyclobacillus* spp. as indicator strains and at 30°C with other indicator strains. The antimicrobial efficacy was determined based on the diameters of inhibition zones.

### Identification of *L. plantarum* YKX

*L. plantarum* YKX was identified according to morphological, biochemical, physiological, and genotypic features. 16S rDNA gene primers, 27F (5′-AGTTTGATCMTGGCTCAG-3′) and 1492R (5′-GGTTACCTTGTTACGACTT-3′) were utilized for PCR amplification. Sangon Biotech Co., Ltd., sequenced the PCR products (Hu et al., 2013), and DNA sequences were compared with the Basic Local Alignment Search Tool (BLAST^1^). The phylogenetic tree was acquired with Mega 7.0 software (Center for Evolutionary Medicine and Informatics, Biodesign Institute, AZ).

### Purification of Plantaricin YKX

Plantaricin YKX was purified by protein precipitation, SP-Sepharose cation-exchange chromatography (80 × 2.0 cm, Sigma, Santa Clara, CA, United States), and reversed-phase high-performance liquid chromatography (RP-HPLC) (Agilent Technologies, Palo Alto, CA, United States). First, *L. plantarum* cells (50 mL) were cultured in MRS (1 L) at 30°C for 24 h. Next, the cell-free supernatants were concentrated to 1/5th of the initial volume using a rotavapor (RV-8V, IKA, Staufen, Germany). After that, ammonium sulfate was added to 50% (*v/v*) saturation and stirred overnight at 4°C. The obtained residue was suspended in 20 mM disodium hydrogen phosphate-citric acid buffer (pH 5.0), loaded on the SP-Sepharose column, and eluted with the same buffer at 0.5 mL/min. Next, the collected fraction showing the highest activity was scanned to obtain the maximal absorption wavelength and then precipitated with 50% aqueous methanol (*v/v*) and vortexed for 1 min. Further, it was fractionated using a Dionex UltiMate 3000 HPLC system with a photodiode array detector coupled with an Agilent HC-C18 column (5 μm, 250 mm × 4.6 mm). A linear gradient of ACN/water (10–95%) over 40 min was used for elution at 0.5 mL/min. The antibacterial activity was determined by agar well diffusion assay using *A. acidoterrestris* DSM3922 as an indicator. The Bradford assay measured the protein concentration.

### Primary Structure Elucidation of Plantaricin YKX

Matrix-Assisted Laser Desorption/Ionization Time of Flight Mass Spectrometry (MALDI-TOF-MS) (autoflex™ speed, Bruker, Germany) was used for determining the molecular masses of plantaricin YKX (Pei et al., 2020). The N-terminal amino acid sequence of plantaricin YKX was obtained by the automated Edman degradation on a Shimadzu PPSQ-21A Protein Sequencing apparatus (Kyoto, Japan) (Pei et al., 2020). Physicochemical properties were determined utilizing bioinformatics tools freely available on https://web.expasy.org/protparam/ (ProtParam tools in Expasy ProtParam, Swiss) and Hyperchem 8.0 software (Hypercube, Gainesville, FL) (Pei et al., 2020).

### Stability of Plantaricin YKX

The effect of temperature (60, 80, or 100°C) on the activity of purified plantaricin YKX was analyzed within a certain time (10, 20, or 30 min) (An et al., 2017). The long-term stability was investigated at 37°C for 2 As and at 4°C for 3 months (Yue et al., 2013). The impact of pH on plantaricin YKX was analyzed by adjusting pH to 2–10 with 1 M HCl and 1 M NaOH solutions and determining residual activities after maintaining at 37°C for 2 h (Yue et al., 2013). The effect of enzymes on the antibacterial activity of plantaricin YKX was determined following incubation with enzymes lipase [pH 7.0, 10 mM phosphate-buffered saline (PBS)], α-amylase (pH 7.0, 10 mM PBS), trypsin (pH 7.5, 10 mM PBS), proteinase K (pH 7.5, 50 mM Tris-HCl), papain (pH 7.5, 10 mM PBS), and pepsin (pH 3.0, 0.1 M HCl) at 37°C for 30 min with their optimal pH and a final concentration of 1.0 mg/mL. The enzymes were inactivated by heating at 100°C for 5 min, and the pH was then adjusted to pH 6.0 (Yue et al., 2013). Plantaricin YKX without any treatments was employed as a control. Agar well diffusion assay was utilized to determine the antibacterial activity (Pei et al., 2020).

### Measuring the Antibacterial Activity

The minimal inhibitory concentration (MIC) of plantaricin YKX toward indicator strains was measured according to the Clinical and Laboratory Standards Institute (2012). Indicator strains in the mid-logarithmic growth phase were adjusted to the OD_600_ of 0.5 and diluted 100-fold with appropriate media. First, the plantaricin YKX solution (1,024 IU/mL) was twofold serially diluted with distilled water. Next, 100 μL each indicator strain suspension and aliquots of plantaricin YKX solution were incubated [1:1 (*v/v*) ratio] in a 96-well plate for 24 h at 45°C with *Alicyclobacillus* spp. as indicator strain and at 30°C for other indicator strains listed in Table 1. The efficacy of nisin (> 90% pure, J & K Chemical Technology) was also determined. As a control, distilled water was added to the indicator strains suspensions. Following incubation, the MICs were determined by measuring the OD_600_ using a microplate reader (SpectraMax 190, Molecular Devices, CA, United States).

**TABLE 1:** Antimicrobial spectrum of *Lactobacillus plantarum* strain YKX and the activity of its bacteriocin.

Microorganisms	Diameter of inhibition zone (mm)	MICs (μg/mL)
Gram-positive bacteria		
*A. acidoterrestris* DSMZ3922	20 ± 2.1	16
*A. acidoterrestris* ^a^	21 ± 1.6	16
*A. acidocaldarius* ^b^	21 ± 1.7	16
*Bacillus subtilis* CICC 10034	20 ± 2.3	16
*B. cereus* CICC 2155	20 ± 1.9	16
*Micrococcus luteus* CICC 10209	13 ± 1.2	32
*Brochothrix thermosphacta* CICC 10509	14 ± 0.7	32
*Clostridium butyricum* CICC 10350	13 ± 1.3	32
*Staphylococcus aureus* CICC 10384	20 ± 2.3	16
*Listeria innocua* CICC 10416	20 ± 1.8	16
*L. monocytogenes* CICC 21529	20 ± 2.2	16
*Lactobacillus helveticus CICC 6024*	31 ± 1.8	4
*Bifidobacterium animalis CICC 6165*	26 ± 2.3	8
*Bifidobacterium bifidum CICC 6071*	25 ± 2.7	8
*Lactobacillus brevis CICC 6239*	32 ± 2.3	4
Gram-negative bacteria		
*Escherichia coli* CICC 10302	7 ± 0.8	64
*Pseudomonas aeruginosa* CICC 21636	−	> highest conc. tested
*Enterobacter cloacae* CICC 21539	−	> highest conc. tested
*Salmonella paratyphi* β CICC 10437	−	> highest conc. tested
Fungal		
*Aspergillus niger* CICC 2124	−	> highest conc. tested
*Candida albicans* CICC 1965	−	> highest conc. tested
*Saccharomyces cerevisiae* CICC 1002	−	> highest conc. tested

*CICC, China Center of Industrial Culture Collection.*

*^a^9 strains of A. acidoterrestris were isolated from the apple garden.*

*^b^3 strains of A. acidocaldarius were isolated from apple juice processing lines. Antimicrobial activity –: there is no inhibition zone.*

### Flow Cytometry

Log phase *A. acidoterrestris* DSM3922 cells were incubated with plantaricin YKX at 0.5×, 1×, or 2× MIC (control: cells treated with PBS) at 45°C for 15 min. Next, they were centrifuged, rinsed, and treated with propidium iodide (10 μg/mL) for 15 min at 45°C (Gut et al., 2011). After removing the unbound stain, the fluorescence intensities were analyzed by an AccuriC6 flow cytometer (BD Biosciences, MI, United States). Forty thousand events were acquired per sample and detected at excitation (488 nm) and emission wavelengths (525 nm).

### Confocal Laser-Scanning Microscopy

Membrane alterations were analyzed using the LIVE/DEAD Bac light bacterial viability kit (Invitrogen, Carlsbad, CA, United States) (Du et al., 2018). Log-phase *A. acidoterrestris* DSM3922 cells were subjected to plantaricin YKX at 1 × MIC at 45°C for specific periods and rinsed with PBS. After that, they were incubated in the absence of light for 15 min at 45°C with SYTO9 and propidium iodide. Fluorescence images were obtained with a PerkinElmer UltraVIEW VoX CLSM system using excitation/emission wavelengths of 490/635 and 480/500 nm for propidium iodide and SYTO9, respectively.

### Endospore Germination

*A. acidoterrestris* DSM3922 endospores were prepared according to Yue et al. (2013). To eliminate the effect of the different living cells of *A. acidoterrestris* on the results which plantaricin YKX might cause over MIC concentration, plantaricin YKX at a sub-lethal concentration (0.5×, 0.7×, or 0.9×, MIC) was added to the pre-prepared spores of *A. acidoterrestris*. Spores with PBS solutions were used as control. Samples were activated by heat shock for 30 min at 70°C in a water bath and 0°C for 15 min in an ice bath. Endospores germination was carried out by transferring the spore suspensions to the fresh AAM medium containing 10 mmol/L alanine and cultured at 45°C. Periodic samples were taken and subjected to Schaeffer and Fulton staining (the spores were stained green, and the bacteria were stained red). The effect of plantaricin YKX on the spore germination rate of *A. acidoterrestris* was assessed by microscopic counting (Pei et al., 2020).

### Guaiacol Production

*A. acidoterrestris* DSM3922 was inoculated in AAM broth containing plantaricin YKX at 0.5 ×, 0.7 × or 0.9 × MIC (control without plantaricin YKX) and cultured at 45°C. Samples were tested for OD_600_ after 24 h. After the samples were centrifuged at 8,000 *g* at 4°C and passed through 0.22 μm filter membrane, the guaiacol content was determined with an analytical HPLC dual pump Shimadzu LC-20 AB system equipped with UltimateXB-C18 column (4.6 × 250 mm, particle size 5 μm, Kyoto, Japan) (Lin et al., 2006). The mobile phase was 30% (*v/v*) acetonitrile in 0.1% (*v/v*) formic acid. The column temperature was maintained at 40°C. The mobile phase flow rate was set at 1 mL/min. The detection wavelength was 275 nm.

### Real-Time Quantitative PCR

To verify the effect of plantaricin YKX on the QS system, Real-Time Quantitative PCR (RT-qPCR) was used to detect the transcription level changes of the gene (*luxS*) encoding a QS signal (Al-2) synthesis enzyme (LuxS) in *A. acidoterrestris*. The mid-logarithmic phase cells of *A. acidoterrestris* were treated with plantaricin YKX at 0.5 ×, 0.7 ×, or 0.9 × MIC. After overnight incubation, RNA was extracted according to the Trizol method.

The reverse transcription reaction system (10 μL) contained: Total RNA 2 μL; dNTP 0.5 μL; Random primers 0.5 μL; and distilled water without RNAase 4 μL. All samples were maintained at 70°C for 5 min and then kept immediately in an ice bath. 5X reverse transcription buffer 2 μL, RNAase inhibitor 0.5 μL, and MMLV reverse transcription enzyme 0.5 μL were added immediately. Thus, the reverse transcription conditions were: 30°C for 10 min, 42°C for 1 h, 70°C for 15 min, and 4°C forever. After the reverse transcription reaction, the DNA templates were diluted 5 times and stored at −20°C until used.

PCR reaction included: upstream primer (10 μM) 0.2 μL, downstream primer (10 μM) 0.2 μL, 2 × Ultra SYBR Mixture 5 μL, ddH_2_O 3.6 μL, DNA templates 1 μL. The PCR reaction parameters were: 95°C for 10 min, 95°C for 15 s, 60°C for 1 min, 40 cycles. Primers (5′-GAGATCTTATGCCATCAGTAGAAAG-3′; 5′-GGTCACCTTTATCCAAACACTTTCTC-3′) were designed according to the gene sequence of *luxS* by the Primer 5.0 software.

### Statistical Analyses

Data were evaluated utilizing the SPSS Statistics 20.0 package, and a significant difference (*P* < 0.05) was obtained using one-way ANOVA. Data were represented as mean ± standard deviation of a minimum of three replicates.

## Results and Discussion

### Isolation and Selection of Bacterial Strain

The strain YKX was chosen for subsequent studies due to its significant inhibition against *Alicyclobacillus* spp. and several other spoilage and pathogenic microorganisms, including Gram-positive and negative bacteria. However, the strain YKX was inactive against fungi, such as *Aspergillus niger*, *Candida albicans*, and *Saccharomyces cerevisiae* (Table 1).

It is noteworthy that the bacteriocin from strain YKX inhibited the growth of Gram-negative bacteria. In contrast, most bacteriocins from LAB, such as nisin, Pediocin PA-1, and plantaricin C, only inhibited Gram-positive bacteria (Gut et al., 2011; Seddik et al., 2017; Pei et al., 2020). The target sites of these bacteriocins were on the cell membrane of Gram-positive sensitive bacteria. Due to the structural differences of the cell membrane, these bacteriocins did not exhibit any antimicrobial activity against Gram-negative bacteria. The relatively narrow antibacterial spectrum is one of the bottlenecks of applying bacteriocins in the food industry. However, some bacteriocins and bacteriocin from strain YKX have displayed antibacterial activity against Gram-negative bacteria in recent years. For example, Du et al.2018 reported that a new bacteriocin, plantaricin GZ1-27, was active against *E. coli* (Du et al., 2018). Lohans et al. (2013) stated that enterocin 7A and 7B could inhibit the growth of Gram-negative bacteria. These bacteriocins (include plantaricin YKX in this study) are mostly proved to have multi-mode of actions against sensitive bacteria, including dissipation of intracellular ATP (Todorov et al., 2011), combination with nucleic acids (Hu et al., 2013), and interaction with cellular metabolism (Chopra et al., 2015; Pei et al., 2020). This multi-mode of action might provide these bacteriocins with a broader inhibitory activity spectrum, even against Gram-negative bacteria (Todorov et al., 2011; Hu et al., 2013).

### Identification of the Strain YKX

**FIGURE 1:**
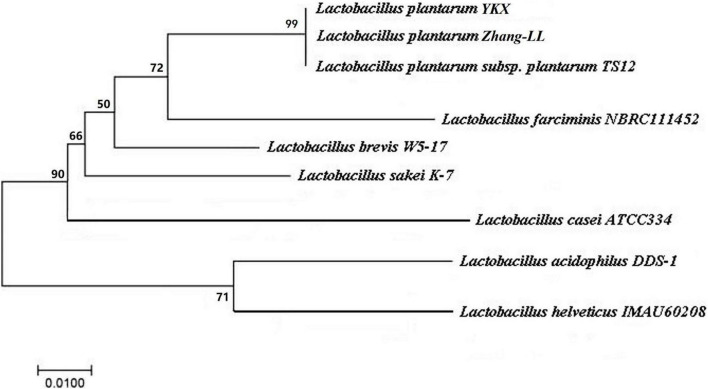
Phylogenetic tree based on 16S rDNA sequences of the isolate.

The strain YKX is a non–spore-forming Gram-positive bacillus. It lacks catalase activity and does not produce gas, illustrating that the strain YKX belongs to homo-fermentative *Lactobacillus*. Additionally, the sugar fermentation test demonstrated that the strain YKX could ferment most of the sugars except rhamnose. The 16S rDNA testing indicated that the strain has been identified as *Lactobacillus plantarum* (Figure 1) and designated as *Lactobacillus plantarum* YKX.

The application of *L. plantarum* in food is well documented. Most studies address its safety profiles (Hu et al., 2013; Algburi et al., 2016; Seddik et al., 2017). Nowadays, *L. plantarum*, probiotic bacteria, is generally recognized as safe. Strain YKX was determined as an *L. plantarum*. This strain may be potentially used as a bacteriocin-producing strain and a starter culture for fermented foods.

### Purification and Sequence Analysis of Plantaricin YKX

**FIGURE 2:**
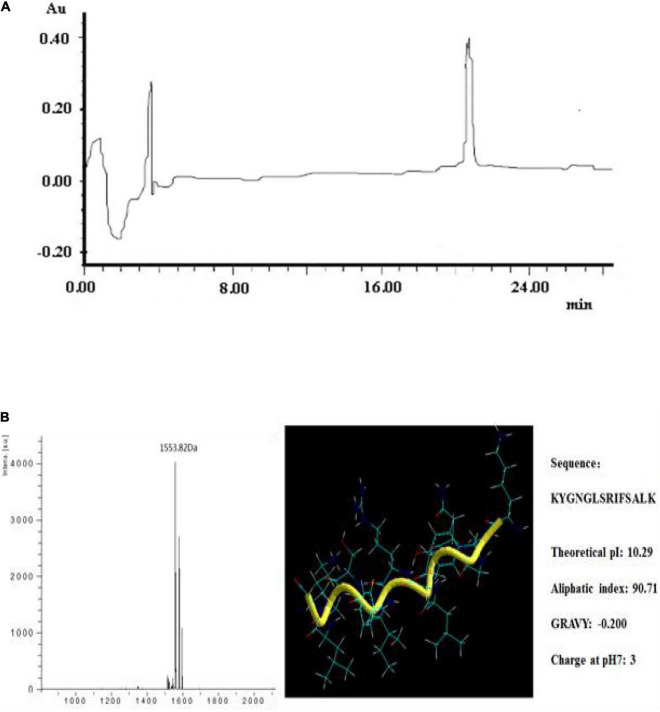
Purification and structural information of plantaricin YKX. **(A)** RP-HPLC analysis of plantaricin YKX; **(B)** MS analysis and theoretical structural information of plantaricin YKX.

The specific activity of plantaricin YKX was augmented to 273.08 IU/mg following ammonium sulfate precipitation. Purification enhanced the activity from 250.00 to 2476.88 IU/mg (7.6-fold increase). A distinct peak exhibiting activity was noticed in the RP-HPLC at 20.8 min (Figure 2A).

The classic three-stage process used herein to purify plantaricin YKX has been successfully employed to purify several bacteriocins from cultures, including plantaricin 163 (Seddik et al., 2017), plantaricin FT259 (Winkelströter et al., 2015), garvicin A (Barragãn et al., 2013), and enterocins 7A and 7B (Lohans et al., 2013). In addition, the industrial production of the well-known bacteriocin, nisin, was also obtained by the standard three-stage process.

The molecular weight (MW) of plantaricin YKX was 1553 Da by MALDI-TOF/MS (Figure 2B). The amino acids determined by N-sequencing were Lys-Tyr-Gly-Asn-Gly-Leu-Ser-Arg-Ile-Phe-Ser-Ala-Leu-Lys (KYGNGLSRIFSALK). The predicted physicochemical properties and the 3D structure of plantaricin YKX are shown in Figure 2B. It is expected to be a random coil conformation. Plantaricin YKX sequence was not similar to that of the other bacteriocins in the NCBI BLAST search.

Most of Class I and Class IIa bacteriocins, such as nisin (3.4 kDa) (Gut et al., 2011), bacteriocin M1-UVs300 (3.3 KDa) (An et al., 2017), and plantaricin C (6.5 KDa) (Yue et al., 2013), have peptide masses greater than 2 kDa. Their primary target is the cell membrane of sensitive bacteria, with a relatively narrow range of activity. Plantaricin YKX has a molecular mass of 1,553 Da. Notably, there are also several small size bacteriocins exhibiting broad-spectrum of activity, such as plantaricin JLA-9 (950 Da) (Zhao et al., 2016) and bacteriocin SLG10 (1,422 Da) (Pei et al., 2020). Small size bacteriocins can readily penetrate the cell membrane and exert different mechanisms of action (Liu et al., 2016). Small molecular weight bacteriocins have consequently attracted research attention.

### Stability of Plantaricin YKX

Plantaricin YKX was heat stable, retaining activity after heating at 60, 80, or 100°C (Table 2). Such thermostability agreed with that of bifidocin A (Liu et al., 2016), plantaricin JLA-9 (Zhao et al., 2016), plantaricin GZ1-27 (Zhao et al., 2016), plantaricin C (Yue et al., 2013), and plantaricin K25 (Wen et al., 2016). Storage of plantaricin YKX at 37°C for up to 14 days or 3 months at 4°C did not alter its antibacterial activity (Table 2). Plantaricin YKX was also stable under acidic, neutral, and slightly alkaline conditions (pH 2.0–8.0); the activity was lost at pH 9.0 (Table 2).

**TABLE 2:** Stability of plantaricin YKX.

Test parameters	Antibacterial ability	
	*A. acidoterrestris* DSM3922 as indicator stain	*E. coli* CICC10302 as indicator stain
Lipase, α-AMYLASE	+	+
Proteinase K, papain, α-chymotrypsin	–	–
Trypsin, pepsin	+	+
60, 80, and 100°C	+	+
37°C for 14 d	+	+
2 months at 4°C	+	+
pH 2–7	+	+
pH 9–10	–	–

*Antimicrobial activity +: there is a clear inhibition zone.*

*Antimicrobial activity –: there is no inhibition zone.*

### Antibacterial Activity of Plantaricin YKX

**FIGURE 3:**
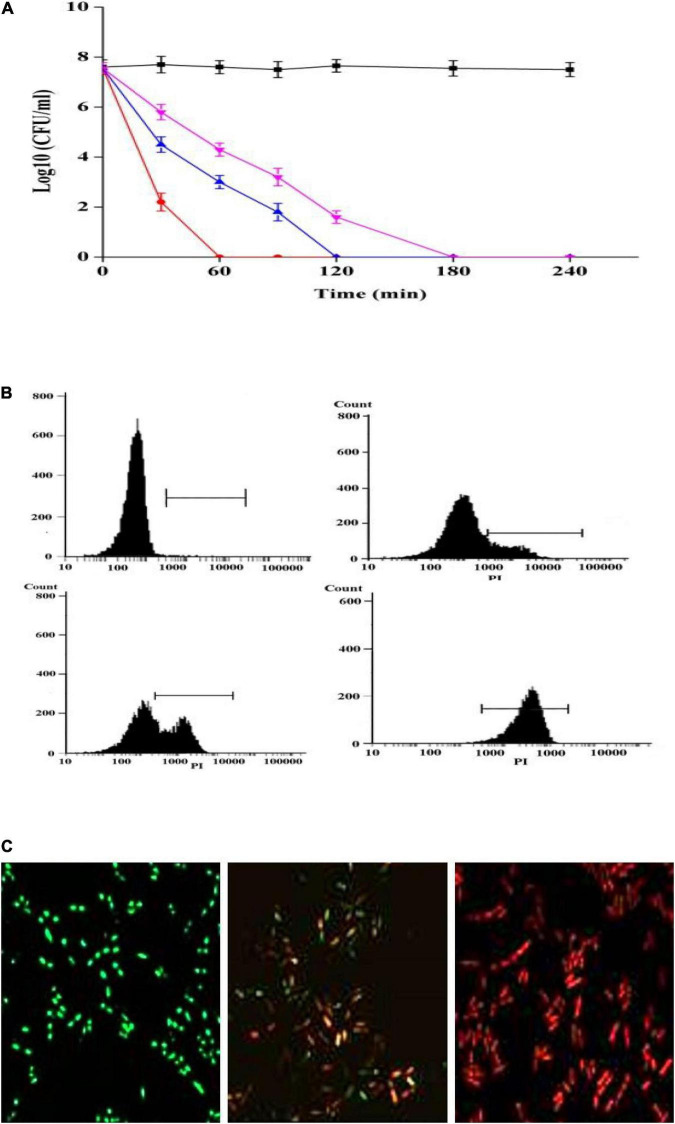
Antibacterial activity of plantaricin YKX against *A. acidoterrestris*. **(A)** Time-killing curves of plantaricin YKX and nisin in *A. acidoterrestris*. ■: control; ▼: 1 × MIC nisin; ▲: 1 × MIC plantaricin YKX; •: 2 × MIC plantaricin YKX; **(B)** Effects of plantaricin YKX on the membrane integrity; treated with plantaricin YKX for 0 × MIC (left up), 0.5 × MIC (right up), 1 × MIC (left down), and 2 × MIC (right down); **(C)** CLSM images of *A. acidoterrestris* cells treated with plantaricin YKX (1 × MIC) for 0 min (left), 30 min (middle) and 60 min (right).

The MIC of plantaricin YKX against *Alicyclobacillus* spp. was 8 μg/mL (Table 1), whereas that of nisin was 16–32 μg/mL (Yamazaki et al., 2000). Analogous outcomes were observed in assessing the kill kinetics assay of *A. acidoterrestris* ATCC 3922 (Figure 3A). After treatment with plantaricin YKX at 2 MIC, the number of *A. acidoterrestris* cells was substantially decreased within 30 min. On the other hand, the MICs of plantaricin YKX and nisin inactivated all cells within 180 min.

To our knowledge, three investigations explored the activity of bacteriocins on *Alicyclobacillus* spp. On this occasion, Grande et al. (2005) have evaluated the extent of membrane damage of four strains of *Alicyclobacillus* by enterocin AS-48. In comparison, Yamazaki et al. (2000) examined the effect of nisin on the outgrowth inhibition of two strains of *A. acidoterrestris* spores. We previously reported the anti *Alicyclobacillus* spectra of bificin C6165 (20/20 strains of *Alicyclobacillus* spp.), bacteriocin RC20975 (18/20 strains), and plantaricin C (17/20 strains) (Yue et al., 2013; Pei et al., 2020). The estimated MIC and time-kill kinetics data suggested the potency of plantaricin YKX against *A. acidoterrestris* in line with these reported bacteriocins but with a broader spectrum (all of these five previously reported bacteriocins were not able to inhibit the growth of Gram-negative bacteria).

Propidium iodide is a nucleus-stain incapable of passing through intact cell membranes but can pass through disrupted membranes. Hence, the quantity of propidium iodide uptake can indicate the degree of membrane damage. Following incubation of *A. acidoterrestris* with plantaricin YKX at 0 ×, 1 ×, and 2 × MIC for 30 min, the proportions of stained PI were 23.5, 37.5, and 91.6%, respectively (Figure 3B). These results indicate that the membrane disruption occurred dose-dependently. Next, we assessed the viability of *A. acidoterrestris* cells incubated with 1 × MIC plantaricin YKX utilizing Live/Dead BacLight staining. *A. acidoterrestris* with intact membranes appeared green due to SYTO. In contrast, those with disrupted membranes appeared red due to propidium iodide entry. Changing from greenish to red fluorescence was detected as the treatment time increased (Figure 3C), which strongly suggested that plantaricin YKX mediated cell membranes’ destruction and eventually led to cell death. Similar results were reported for bacteriocins, such as nisin (Yamazaki et al., 2000), plantaricin K25 (Wen et al., 2016), bifidocin A (Liu et al., 2016), and enterocin 7A and 7B (Lohans et al., 2013).

### Effect of Plantaricin YKX on Spore Germination, the Production of Guaiacol, and Transcription of luxS

**FIGURE 4:**
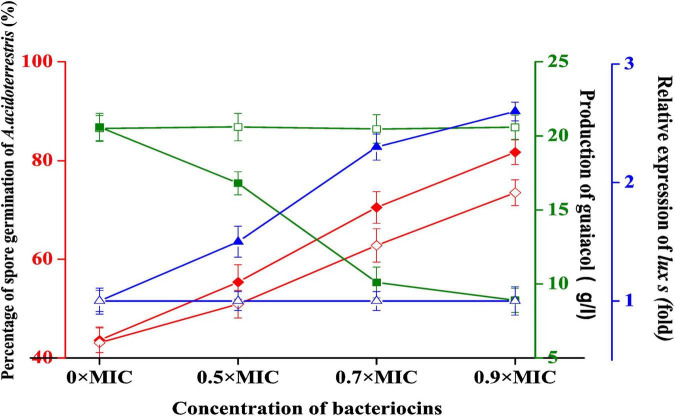
Effect of plantaricin YKX and nisin on *A. acidoterrestris*. ◆ Effect of plantaricin YKX on the spore germination; ◆ the impact of nisin on the spore germination; ■ and the impact of plantaricin YKX on guaiacol production. □ the impact of nisin on the guaiacol production; ▲ effect of plantaricin YKX on the relative expression of *luxs*, △ the effect of nisin on the relative expression of *luxs.*

The germination rate of *A. acidoterrestris* spores without plantaricin YKX treatment was 43.6%, 2 h post-germination treatment. In contrast, the treatment with plantaricin YKX at 0.5 × MIC, 0.7 × MIC, and 0.9 × MIC had a 55.4, 70.5, and 81.7% germination rate, respectively (Figure 4). A similar trend was also noticed in nisin. These results indicate that plantaricin YKX and nisin could germinate the spores of *A. acidoterrestris* into vegetative cells. The spores can survive pasteurization; however, vegetable cells were not. So the effect of sterilization of *Alicyclobacillus* by pasteurization might be improved by combining it with plantaricin YKX.

In apple, orange, and grapefruit juices, the vegetative cells of A. acidoterrestris are converted into endospores under highly acidic pH values of 3.4–3.9. Heat-resistant spores have *D*-values of approximately 8 min at 97°C and are not damaged under pasteurization, generally used in the juice industry. In this context, Yamazaki et al. (2000) stated that nisin could not inhibit the spore germination of *A. acidoterrestris*; however, it could significantly reduce the heat-resistant spores. Similar reports were also seen with bificin C6165, bacteriocin RC20975 (Yue et al., 2013), and plantaricin C (Pei et al., 2020). This study states that plantaricin YKX and nisin would affect the spore germination of *A. acidoterrestris*, explaining the phenomenon. This is because these bacteriocins could induce the germination of *A. acidoterrestris* spores, converting them to vegetative cells with relatively low heating tolerances.

After treatment with plantaricin YKX at 0.5 × MIC, 0.7 × MIC, and 0.9 × MIC, the production of guaiacol by *A. acidoterrestris* decreased from 20.6 to 16.8, 10.1, and 8.9 μg/L, respectively, compared with the control (Figure 4). Thus, unlike nisin, plantaricin YKX may inhibit the biosynthesis of guaiacol in a dose-dependent manner.

Guaiacol is one of the main spoilage compounds produced by *Alicyclobacillus* spp. in acidic juices. Concentrations of guaiacol at ppm levels may cause taste impairments and turbidity (Yamazaki et al., 2000; Lin et al., 2006; Du et al., 2018). Like plantaricin YKX, several bacteriocins, such as bacteriocin Ent35-MccV (Acuna et al., 2012), plantaricin GZ1-27 (Du et al., 2018), and plantaricin JLA-9 (Zhao et al., 2016) are also showed inhibitory effects on the secondary metabolic compounds of the sensitive bacteria. Compared with nisin, one of the most famous bacteriocins, plantaricin YKX (Yamazaki et al., 2000), was not only able to inhibit the growth of *Alicyclobacillus* spp. but also decreases the production of guaiacol.

The electrophoresis of RT-PCR products is shown in Supplementary Figure 1. The relative expression of the luxS gene was calculated based on the fluorescence intensity of the bands (Supplementary Figure 1). The expression of the *luxS* gene increased significantly after being co-cultured with plantaricin YKX at 0.5 × MIC, 0.7 × MIC, and 0.9 × MIC, suggesting that plantaricin YKX can affect the QS system of *A. acidoterrestris* (Figure 4). Nisin did not show a similar function (Supplementary Figure 1).

QS systems refer to the regulation system in which bacteria spontaneously produce and release specific signaling molecules that regulate various biological behaviors, such as toxin production, biofilm formation, antibiotic production, spore formation, and fluorescence production in the microbial community by sensing their concentration changes. At present, the research on QS systems has become a hot topic in the field of microbiology. QS systems are categorized into three types: (1) AHL lux I/Lux R system in the Gram-negative bacteria; (2) Oligopeptide mediated two-component sensing system in Gram-positive bacteria; and (3) LuxS/Al-2 dependent QS system.

A variety of QS systems have been identified in the same bacterial species. For example, the LuxS/Al-2 dependent QS system exists in Gram-positive and negative bacteria, and Al-2, a universal signaling molecule, participates in exchanging information. Because bacteria live together in communities, the LuxS/Al-2 dependent QS system and Al-2, a ubiquitous signaling molecule, are crucial for bacteria to form a relatively stable ecological environment with a proportional number of bacteria and functional divisions. In addition, researchers found that in many bacteria, some biological functions, such as antibiotic synthesis, virulence factor expression, biofilm formation, bioluminescence, and bacteriocin synthesis, are regulated by LuxS/Al-2 dependent QS system.

LuxS protein is the key enzyme of Al-2 production. The *luxS*-coding genes exist in a variety of Gram-positive and -negative bacteria and are highly conservative. All Al-2 is a by-product of the methyl cycle. LuxS protein is an essential enzyme for the production of Al-2 and plays a vital role in the metabolism of the methyl cycle. Because the low concentration of natural extracellular Al-2 molecule in the CFS of *Alicyclobacillus* spp. is not conducive for analysis and detection. So, we carried out exploratory research on LuxS protein to evaluate the effect of plantaricin YKX on the expression of the *luxS* gene. The results showed that plantaricin YKX could promote the expression of the *luxS* gene.

Concerning the mode of action of bacteriocins, most articles were focused on the inhibition mechanism of bacteriocin on sensitive bacteria. Few reports were investigating the effect of bacteriocin on the QS system of sensitive bacteria. The results in this study suggested that not all bacteriocins affect the QS system of the susceptible bacteria (plantaricin YKX can, but not nisin). For plantaricin YKX, the ability to influence the spore germination and production of guaiacol might be attributed to its ability to regulate the QS system of *A. acidoterrestris.* However, for nisin, the ability to affect the spore germination of *A. acidoterrestris* might be ascribed to other reasons. Thus, the mode of action of bacteriocins appears to be diverse despite having similar effects on sensitive bacteria. Similar trends were proposed for plantaricin K25 (Wen et al., 2016), bifidocin A (Liu et al., 2016), plantaricin 163 (Hu et al., 2013), and plantaricin JLA-9 (Zhao et al., 2016).

## Conclusion

In summary, bacteriocin-producing strain *L. plantarum* YKX was isolated and identified by 16S rDNA. Its bacteriocin, plantaricin YKX, showed good antibacterial activity against *Alicyclobacillus acidoterrestris*. Plantaricin YKX was also active against Gram-negative bacteria (*E. coli*). A 14-amino acid peptide (Lys-Tyr- Gly- Asn- Gly- Leu- Ser- Arg-Ile-Phe-Ser-Ala-Leu-Lys); active against *A. acidoterrestris* at the “cell membrane damage” levels. The plantaricin YKX was also able to decree the secretion of guaiacol. Therefore, it is warranted to study the mechanism of action of plantaricin YKX and develop its applications in food biopreservation.

## Data Availability Statement

The original contributions presented in the study are included in the article/Supplementary Material, further inquiries can be directed to the corresponding author/s.

## Author Contributions

JP: in charge of the whole program organization, operating, and funding supporting, and manuscript writing. WJ: in charge of the bacteriocin producer isolation. JW and YH: in charge of the experiments operating and data collection and data analysis. XL: in charge of the antibacterial activity part. HZ and YZ: in charge of the manuscript review. AA: in charge of the manuscript review and edit. All authors contributed to the article and approved the submitted version.

## Conflict of Interest

The authors declare that the research was conducted in the absence of any commercial or financial relationships that could be construed as a potential conflict of interest.

## Publisher’s Note

All claims expressed in this article are solely those of the authors and do not necessarily represent those of their affiliated organizations, or those of the publisher, the editors and the reviewers. Any product that may be evaluated in this article, or claim that may be made by its manufacturer, is not guaranteed or endorsed by the publisher.

## Footnotes

1
https://www.ncbi.nlm.nih.gov/

## Supplementary Material

Supplementary Figure 1 Electrophoresis of PCR products. Marker from down to top is 2000, 1000, 750, 500, 250, and 100; Band #1, #3, #5: amplification of luxS in cells treated with nisin. Band #2: amplification of luxS in the cells treated with 0.5 MIC plantaricin YKX. Band #4: amplification of luxS in the cells treated with 0.7 MIC plantaricin YKX. Band # 6: amplification of luxS in the cells treated with 0.9 MIC plantaricin YKX.
