# Supplementary figures and images for: Purification and Characterization of Plantaricin YKX and Assessment of Its Inhibitory Activity Against Alicyclobacillus spp
Source: Front Microbiol. 2021 Dec 9;12:783266. doi: 10.3389/fmicb.2021.783266 (PMC8696185; doi:10.3389/fmicb.2021.783266)

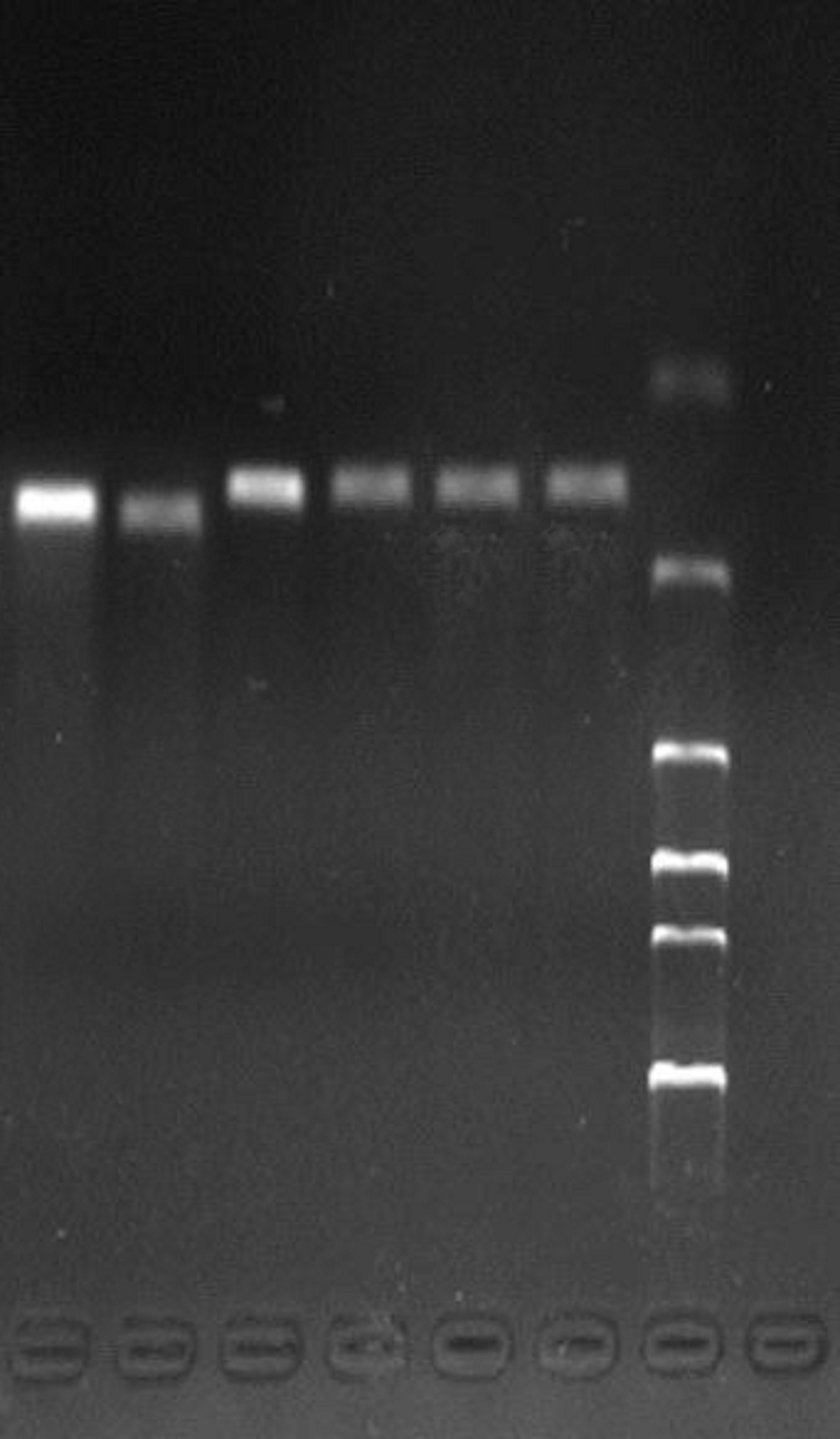

Supplement: Supplementary Figure 1 — Electrophoresis of PCR products. Marker from down to top is 2000, 1000, 750, 500, 250, and 100; Band #1, #3, #5: amplification of luxS in cells treated with nisin. Band #2: amplification of luxS in the cells treated with 0.5 MIC plantaricin YKX. Band #4: amplification of luxS in the cells treated with 0.7 MIC plantaricin YKX. Band # 6: amplification of luxS in the cells treated with 0.9 MIC plantaricin YKX. [file Image_1.jpg]
